# Stand-by emergency treatment (SBET) of malaria in Spanish travellers: a cohort study

**DOI:** 10.1186/s12936-018-2304-7

**Published:** 2018-04-02

**Authors:** Pietro Ferrara, Cristina Masuet-Aumatell, Fernando Agüero, Josep Maria Ramon-Torrell

**Affiliations:** 1 Department of Experimental Medicine, University of Campania “Luigi Vanvitelli”, 5, Via Luciano Armanni, 80138 Naples, Italy; 2 Bellvitge Biomedical Research Institute (IDIBELL), Preventive Medicine Department, University Hospital of Bellvitge, Feixa Llarga s/n, 08907 L’Hospitalet de Llobregat, Catalonia Spain; 3 Clinical Science Department, School of Medicine, University of Barcelona, Feixa Llarga s/n, 08907 L’Hospitalet de Llobregat, Catalonia Spain

**Keywords:** Malaria, Malaria prevention, Standby emergency treatment, Travel medicine, Tropical disease, Prophylaxis adherence, Travellers

## Abstract

**Background:**

Among strategies for malaria prevention, stand-by emergency treatment (SBET) is a possible approach, but scarce evidences exists investigating travellers’ adherence and behaviours toward its use; therefore, the presented study aimed to determine travellers’ compliance toward the SBET when prescribed in travel clinics.

**Methods:**

A prospective cohort study was performed at the Travel Health Clinic of the Hospital Universitari de Bellvitge, Barcelona, Spain, during 2017. The research was planned on survey-based design, using pre- and post-travel questionnaires.

**Results:**

In the study period, of 5436 subjects who attended the HUB Travel Medicine Clinic, 145 travellers to malaria-endemic areas were prescribed SBET, and all patients agreed to participate in the study by completing the pre-travel questionnaire. Approximately half the participants were women (n = 75, 51.7%), and the median age of all travellers was 29 years (range 13–57), mainly travelling to South-East Asia (n = 69, 47.6%), with Indonesia and the Philippines as the most popular destinations. The length of travels had a median duration of 29 days (range 10–213). Of the recruited participants, 98 replied to the online post-travel survey, reaching a response rate of 67.6%. A total of 62.2% of travellers to which SBET was prescribed did not buy and carry drugs while travelling abroad. No participants’ baseline or travel characteristic was shown to be significantly associated (p > 0.05) with this behaviour. Four women (4.1%) experienced fever and self-administered SBET, without seeking medical attention. No malaria cases were observed.

**Conclusions:**

This cohort study addressed travellers’ adherence and behaviour toward SBET, highlighting an incorrect use of the emergency treatment in case of presumptive malaria symptoms. This should be taken into account during pre-travel consultation, since the success of this strategy for malaria prevention depends on travellers’ strong adherence to it.

## Background

Malaria prophylaxis differs substantially from traveller to traveller and from region to region, even within a single country. This variability is a function of national recommendations, intensity of transmission, predominance of *Plasmodium* species and characteristics of the travel, such as destination, itinerary, duration, season and type of travel [1, 2]. Stand-by emergency treatment (SBET) is an approach based on traveller self-treatment to be used when a possible malarial infection could occur while overseas and a prompt medical evaluation is not possible because the traveller is staying in a remote area [3]. SBET should be presumptively self-administered when access to medical care is not available in less than 24 h and the traveller develops a febrile illness consistent with malaria. The traveller should be advised that SBET is a temporary measure for suspected malaria. It is not counterfeit, and it will not interact adversely with the traveller’s other medicines, but a prompt medical evaluation remains imperative [3, 4].

The SBET prescription associated with mosquito avoidance measures is due to a risk-benefits analysis, since the incidence of adverse effects of chemoprophylaxis could outweigh the attack rate of malaria in areas at low risk of transmission [5, 6]. Moreover, the development of further anti-malarial drug resistances, associated with medication intake, and the presence of counterfeit medication in remote areas also foster SBET use [7].

The World Health Organization (WHO) provides general guidelines that travellers carrying SBET should observe, such as to consult a physician immediately if fever occurs 1 week or more after entering an endemic area, to start the SBET when medical care is not available within 24 h of the onset of fever and then to seek medical care as soon as possible for a complete evaluation and to exclude other illnesses and to use antipyretics to lower fever and reduce vomiting due to anti-malarial drugs [5].

Despite these guidelines, the medical community is currently debating the concept of SBET [8–10], and the available literature is partial insufficient to draw firm conclusions. Moreover, regarding the topic of travellers’ adherence to SBET, very little has been published [11–14]; hence, the aim of this study was to determine travellers’ compliance with prescribed SBET.

## Methods

### Study design

A prospective cohort study was performed in a travel clinic among travellers to areas at low risk for malaria who consecutively presented to the clinic in 2017 and agreed to be questioned about treatment use. Travellers who had completed their journey after December 31, 2017 were excluded from the study. The information was gathered through two questionnaires: one that was completed face-to-face prior to the trip and during the medical visit (*pre*-*travel questionnaire*) and another that was completed by email from 2 to 3 weeks after the expected return date (*post*-*travel questionnaire*).

### Setting

The study was conducted at the Travel Health Clinic at the Hospital Universitari de Bellvitge, in Barcelona, Spain.

### Participants

Adults seeking medical advice at the travel clinic, between January 2017 and December 2017, before travelling to areas where SBET could be prescribed, who agreed to participate in the study and to be contacted after the travel were included. SBET was prescribed to travellers who fulfilled the following indications: accepted SBET prescription and travelled to areas at low risk for malaria transmission, regardless of purpose of travel and duration; long-term travellers expected to be scarcely compliant to chemoprophylaxis; travellers without a definite route (such as backpackers). SBET was also proposed to travellers who refused chemoprophylaxis. Individuals who met the following criteria were excluded: people with severe renal impairment (creatinine clearance < 30 mL/min), pregnant women, women breastfeeding infants weighing < 5 kg, those not willing to participate or those who refused SBET prescription.

E-mail addresses were registered to contact the individuals and conduct the follow-up.

The participation was voluntary, participants were not offered any financial incentive and they were informed about their right to withdraw at any time without penalty. The institutional ethical review board (University Hospital of Bellvitge) approved the study protocol and informed consent. All participants provided written informed consent. Confidentiality was maintained by omitting any personal identifying information from data collection.

### Research instruments and outcomes measures

Participants were provided with information about malaria and its prevention measures, and instructed regarding SBET use. A pack of 12 tablets containing atovaquone 250 mg and proguanil 100 mg was prescribed to each of them. Implementation of other effective interventions to avoid mosquito bites, such as frequent repellent use, appropriate clothing, insecticide residual spraying or sleeping under a bed net was also recommended.

For this survey-based prospective cohort study, two structured administered questionnaires were designed, as pre- and post-travel interviews. Both questionnaires were initially tested in a convenience sample of travellers to evaluate the design, clarity and comprehensibility of the items. To estimate the consistency of the responses, some probe questions were used. Based on respondents’ feedback, the initial version was amended, and some minor rewording of the questions was incorporated to simplify and improve the final version for clarity.

The questionnaires were administered by a trained medical doctor face-to-face and by e-mail 2–3 weeks after expected date of return by the same trained medical doctor. Additionally, checks of the Electronic Medical Records HC3 System [15] were performed to investigate travel-related health problems in the study population, as well as visits to GPs or emergency departments.

The pre-travel questionnaire assessed travellers’ socio-demographic characteristics, collected as follows: age, gender, self-reported medical history (comorbidities, current pharmacological treatments) and information about travel, such as country, itinerary, duration and purpose. Previous use of anti-malarial drugs and if they had ever experienced any secondary adverse effects (AE) were also investigated.

Participants were also asked for their e-mail address to be contacted for completion of the post-travel questionnaire, which was delivered to all of them via professional online survey software (Google^®^ Forms) between 2 and 3 weeks after their returns. All travellers received an email inviting them to complete the survey, accessible via an embedded URL link. Non-respondents received a reminder 2 weeks later. A clear preliminary statement provided information to the cohort about the study and instructions and allowed participants to confirm their own informed consent to participate in the survey. The second research instrument, designed for capturing information about participants’ behaviours toward SBET use, comprised a question to investigate whether travellers carried anti-malarial medication during travel abroad. When participants answered “Yes”, they were invited to provide information about the potential self-administration of the prescribed prophylaxis, the felt symptoms (fever, headache, chills, etc.), how long they self-administered drugs, any experienced AEs, and how fast they searched for medical assistance, if it was available.

### Data analysis

Continuous variables were expressed as median and range; categorical variables were described as number and percentage. Due to their non-normal distribution (Kolmogorov–Smirnov test p value < 0.05), a Mann–Whitney U test was used to assess differences between medians, and Chi square (*χ*^2^) or Fisher’s exact tests were used when needed to assess differences between categorical variables. All statistical tests were two-tailed, and a *p* value ≤ 0.05 was considered statistically significant. Data were analysed using SPSS statistical software v. 21.0 (IBM SPSS Statistics for Windows, Armonk, NY, USA).

## Results

**Fig. 1:**
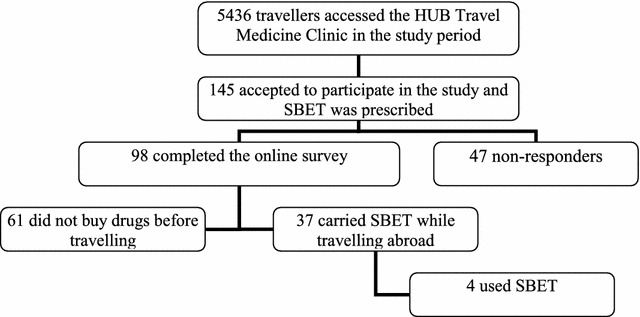
Flow chart of number of participants in the study

From January to December 2017, 5436 travellers presented to the HUB Travel Medicine Clinic, and 1025 intended to travel to malaria-endemic areas, being prescribed SBET in 14.1% of cases (n = 145) as a reliable malaria self-treatment when the traveller planned to stay in remote areas with low risk for disease transmission. All of them agreed to participate in the study by completing the pre-travel questionnaire. A flow-chart of participants can be seen in Fig. 1.

The main characteristics of the study population and travel are listed in Table 1. Approximately half the participants were women (n = 75, 51.7%), with a median age of 29 years (range 13–57), without previous use of anti-malarial drugs (n = 129, 89.0%) travelling to Southeast Asia (n = 69, 47.6%), with Indonesia and the Philippines as the most popular destinations. Also, 10 subjects (6.9%) travelling to Africa were prescribed SBET, despite general recommendations indicating chemoprophylaxis for Sub-Saharan regions. Of this sub-group, 8 had planned to travel to Ethiopia (n = 5) and South Africa (n = 3) and were prescribed with SBET because of the long length of stay or of the absence of a clear itinerary with an unpredictable malaria risk, while the other 2 travellers (going to Ethiopia and Cameroon) refused traditional chemoprophylaxis. The median duration of travel was 29 days (range 10–213). No statistically significant differences (p value > 0.05) were observed above baseline characteristics between patients who answered the post-travel questionnaire and the missing ones.

**Table 1:** Characteristics of study population (*n *=145)

Gender	
Male	70 (48.3%)
Female	75 (51.7%)
Age (in years)^a^	29 (13–57)
Area of destinations	
Africa	10 (6.9%)
India and Nepal	17 (11.7%)
Southeast Asia	69 (47.6%)
Central America	19 (13.1%)
South America	30 (20.7%)
Duration of travel (in days)^a^	29 (10–213)
Previous use of anti-malarial drugs (atovaquone/proguanil)	16 (11.0%)
AE	2 (12.5%)

^a^Variables summarized by median and range

Among travellers who had used atovaquone/proguanil as anti-malarial chemo-prophylaxis (n = 16) in previous travels, 12.5% of them (n = 2) were women of 50 years old (yo) and 26 yo who reported experiencing nausea and vomiting as AEs.

Of the recruited participants, 98 also completed the online survey for a total response rate of 67.6%; of those, only 37 (37.8%) actually carried anti-malarial drugs while travelling abroad, while 61 travellers to which SBET was prescribed did not buy the drugs before travelling. No participants’ baseline or travel characteristic was shown as significantly associated (p value > 0.05) with this behaviour.

The analysis of the post-travel questionnaires showed that 94 subjects (95.9%) did not declare fever or other health problems during or after the travel. Three women (4.1%) experienced fever only, and a fourth complained of fever and diarrhoea: all of them declared SBET self-administration more than 7 days after entering the endemic area; the 4 users did not seek medical care nor quicken their travel (Table 2). Furthermore, 2 of them reported implementing SBET during 3 days; one also declared general malaise and nausea after atovaquone/proguanil consumption.

**Table 2:** Description of SBET users (*n *=4)

	Age and sex	Travel destinations	Travel duration (in days)	Experienced symptoms	SBET administration period (in days)	SBET adverse effects	Consulting medical personnel after SBET use
1	22/F	Thailand, Laos, and Vietnam	50	Fever	3	General malaise, nausea	No
2	27/F	Indonesia	45	Fever	3	None	No
3	28/F	India	48	Fever and diarrhoea	*	None	No
4	34/F	Ethiopia	9	Fever	*	*	No

*SBET* Stand-by emergency treatment

* Missing information

The consultation of regional Electronic Medical Records HC3 System [15] of all participants confirmed no malaria cases, nor other travel-related health problems in the rest of the study population. No visits to general practitioners (GPs) or emergency departments were observed in the study and follow-up periods.

## Discussion

This cohort study offers a first insight into travellers’ use of the SBET in Spain.

Because of travel characteristics, foremost the median length of stay of 29 days, staying in remote areas with low intensity of malaria transmission and a scarce number of imported cases from these areas, the study group was selected as a candidate of SBET strategy instead of anti-malarial chemoprophylaxis.

The most noticeable data yielded through the online post-travel survey is about the consistent number of travellers who did not even carry atovaquone/proguanil while travelling abroad, with two-thirds of them (62.2%) not following medical prescriptions. This behaviour may be explained by the idea that going to areas with low risk for malaria infection falsely reassures travellers [16], leading them to believe it is unnecessary to buy drugs themselves. In this behavioural domain, there were no differences associated with travellers’ demographic profiles or travel characteristics, presenting it as a widespread perception [11]. This aspect represents an unpredictable widespread behaviour among travellers that should be taken into account during pre-travel medical care. These findings foster the idea that more studies are needed to investigate this domain, as well as the implementation of instructive programmes designed to improve the level of compliance to SBET recommendations among travellers.

While it is well known that a large number of people travel without a pre-travel medical consultation [17, 18], a major interest of such a cohort is the relevant number of travellers that still travel without any protection against malaria, although they attended a specific travel clinic where they were informed about risks and received a medical prescription of SBET that could be collected from a pharmacy for less than € 5.

The vast majority of the respondents (95.9%) declared that they did not experience any possible malarial symptoms during travel. Only 4 responders took drugs because of the onset of fever without other symptoms for 3 women, and of fever and diarrhoea in a fourth user. Two SBET users also reported to have complied with the dosage of 4 tablets taken on a daily basis, during 3 days. Not all users sought medical attention nor interrupted their travel. None of these was confirmed as a malaria case from the consultation of regional medical records during the follow-up period.

Even if only represented by 4 travellers, the portion of SBET users (4.1%) in this study is in concordance with results reported by other published studies [11–14]. However, a general scarce level in the application of medical advice was shown in this cohort, since these interviewees detailed an incorrect administration of the SBET regimen. This highlights two relevant issues, regarding the information provided to travellers during pre-travel care, as well as the risk of exposure to drug AEs due to their erroneous use. The use of a strong dose of atovaquone/proguanil in a short period of time increases the likelihood of AEs and toxicity, to which travellers might be needlessly exposed, such as the 22-yo user that also reported general malaise and nausea as atovaquone/proguanil AEs. Also alarming is travellers’ perception of the emergency treatment as an alternative for medical care, although guidelines strongly recommend immediate medical consulting.

Prescription of this prevention strategy is currently increasing, with a natural change from chemoprophylaxis to SBET [19], due to travellers’ preferences for SBET itself more than traditional prophylaxis [20]. Indeed, several arguments support the concept of SBET in those areas with a low risk transmission: (1) the use of SBET, instead of chemoprophylaxis, reduces the likelihood of the development of AEs of the medication; (2) the developing of drug resistances and the presence of counterfeit anti-malarial drugs in these areas also lead healthcare professionals to indicate emergency treatment; (3) some travellers’ characteristics represent situations in which this option can be considered, such as travellers without a definite itinerary, short-stay travellers (such as those who travel for professional purposes) and those who move to remote areas or areas devoid of medical facilities.

While the smooth utility of SBET is ensured if instructions for use are followed, as this research showed, SBET is not a reliable choice for most travellers due to misuse.

Due to this lack of strong scientific evidence regarding the effectiveness of prophylaxis regimens other than traditional chemoprophylaxis, the current UK guidelines for malaria prevention in travellers place emphasis only on the implementation of interventions to avoid exposure, such as precautions against mosquito bites (frequent repellent use, appropriate clothing, insecticide residual spraying, sleeping under a bed net) or seeking medical advice as soon as symptoms develop, without the use of a specific anti-malarial [21].

The main limitations of the study are as follows. First, the number of travellers who visited the clinic to whom SBET was prescribed was limited as a real-world study; for this reason, and due to the likely multi-factoral nature, it was not possible to assess the barriers to their mis-implementation of pre-travel recommendations. Second, there is potential bias due to the use of a traceable and not anonymous online survey, where respondents could be influenced by researchers’ expectations or hide the actual incorrect behaviours. Consultation of regional medical records likely softened this limitation, even though participants may not have sought medical care for minor health problems, leading to underestimation of the actual incidence of travel-related health problems.

Despite these limitations, since the participants in this study represented the standard travel population, it is possible to state that the selected cohort is representative of the target population. Moreover, the enrolment of a consecutive cohort reduces problems of selection and participant bias.

## Conclusions

In conclusion, the findings addressed travellers’ adherence and behaviours toward SBET, expressing an incorrect use of the emergency treatment in cases of presumptive malaria symptoms. Substantial concerns about SBET itself as well as on the need to design and implement proven prevention measures for malaria arise from this information, and they should be taken into account during pre-travel consultation, since the success of this strategy for malaria prevention depends on travellers’ strong adherence to it.

## Abbreviations

SBET: standby emergency treatment
WHO: World Health Organization
AE: secondary adverse effects
HUB: Hospital Universitari de Bellvitge, L’Hospitalet de LLobregat, Spain
